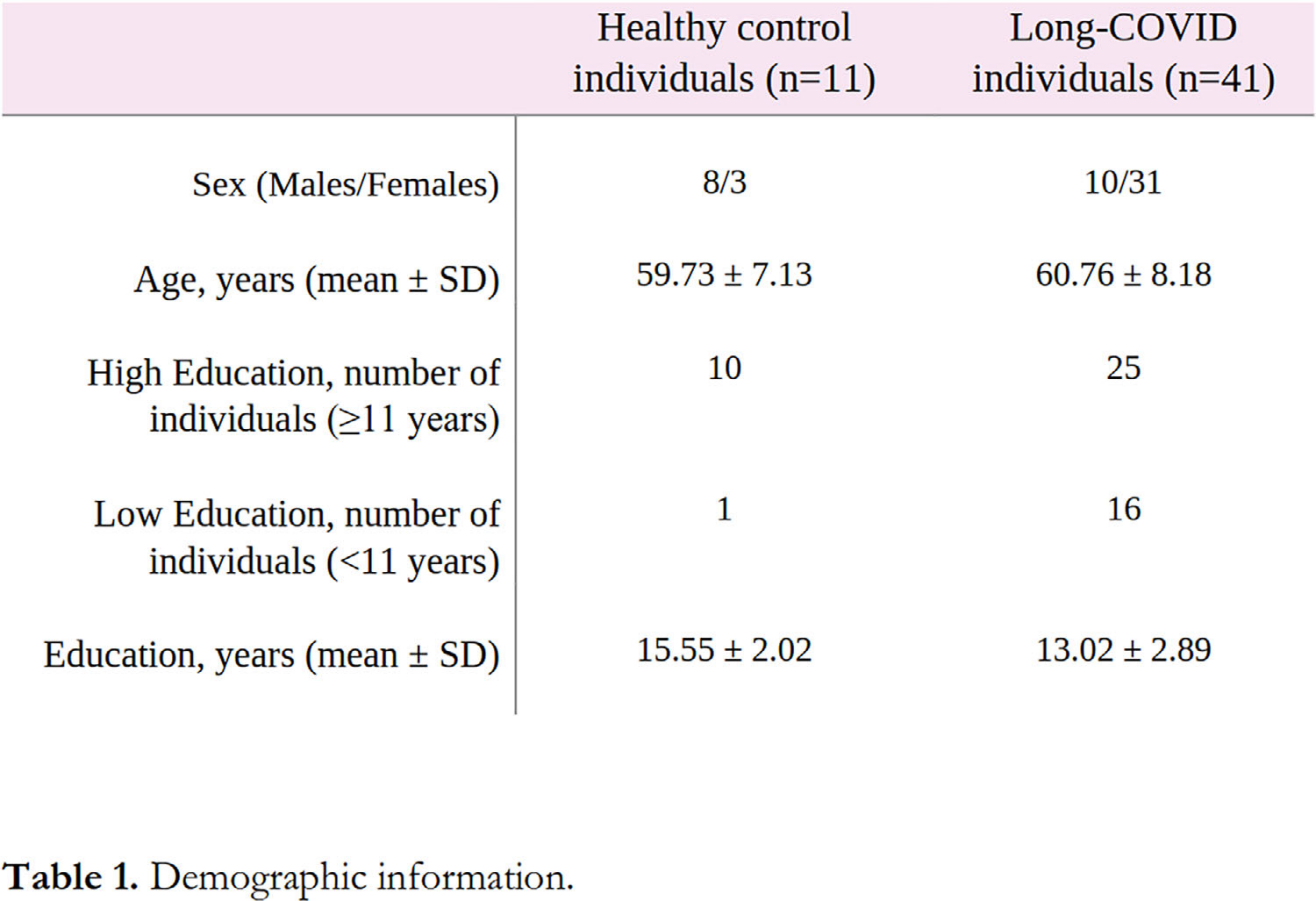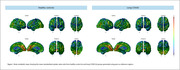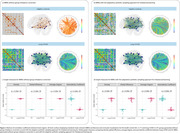# Metabolic Hyperconnectivity in Underrepresented Individuals with long‐COVID

**DOI:** 10.1002/alz.094053

**Published:** 2025-01-09

**Authors:** Luiza Santos Machado, Maiele Dornelles Silveira, Marco Antônio de Bastiani, Guilherme Povala, Wyllians Vendramini Borelli, Joana Emilia Senger, Ana Paula Bornes da Silva, João Pedro Uglione da Ros, Guilherme Bastos de Mello, Arthur Viana Jotz, Matheus Fakhri Kadan, Guilherme G. Schu Peixoto, Graciane Radaelli, Tharick A. Pascoal, Pedro Rosa‐Neto, Cristina Sebastião Matushita, Ricardo Benardi Soder, Artur Francisco Schumacher‐Schuh, Diogo O. Souza, Mychael V Lourenco, Daniele de Paula de Paula Faria, Artur Martins Coutinho, Jaderson Costa da Costa, Débora Guerini de Souza, Eduardo R. Zimmer

**Affiliations:** ^1^ Universidade Federal do Rio Grande do Sul, Porto Alegre, Rio Grande do Sul Brazil; ^2^ Federal University of Rio Grande do Sul, Porto Alegre, Rio Grande do Sul Brazil; ^3^ University of Pittsburgh, Pittsburgh, PA USA; ^4^ Memory Center, Hospital Moinhos de Vento, Porto Alegre, RS Brazil; ^5^ Federal University of Rio Grande do Sul, Brazil, Porto Alegre, RS Brazil; ^6^ Lutheran University of Brazil, Canoas, Rio Grande do Sul Brazil; ^7^ Pontifícia Universidade Católica do Rio Grande do Sul, Porto Alegre, Rio Grande do Sul Brazil; ^8^ Federal University of Rio Grande do Sul, Porto Alegre, RS Brazil; ^9^ Brain Institute, RS, Porto Alegre, Rio Grande do Sul Brazil; ^10^ Douglas Mental Health University Institute, Montreal, QC Canada; ^11^ Brain Institute of Rio Grande do Sul (BraIns), PUCRS, Porto Alegre, RS Brazil; ^12^ Pontifical Catholic University of Rio Grande do Sul, PORTO ALEGRE, RIO GRANDE DO SUL Brazil; ^13^ HCPA, Porto Alegre, Rio Grande do Sul Brazil; ^14^ Universidade Federal do Rio Grande do Sul, Porto Alegre Brazil; ^15^ Institute of Medical Biochemistry Leopoldo de Meis, Federal University of Rio de Janeiro, Rio De Janeiro, Rio de Janeiro Brazil; ^16^ University of São Paulo Medical School, São Paulo, São Paulo Brazil; ^17^ Brain Institute of Rio Grande do Sul ‐ Pontifícia Universidade Católica do Rio Grande do Sul, Porto Alegre, Rio Grande do Sul Brazil

## Abstract

**Background:**

Long‐COVID is characterized by persistent symptoms post‐infection with SARS‐CoV‐2. This condition includes neurological manifestations and has been proposed as a potential risk factor for the development of dementia. Individuals presenting with dementia due to Alzheimer's disease have dysfunctional brain metabolism, including metabolic brain network (MBN) hypoconnectivity. However, whether long‐COVID alters brain metabolic architecture remains elusive. Here, we aimed to evaluate the brain metabolic connectivity in a Brazilian cohort of individuals presenting with long‐COVID.

**Method:**

[18F]FDG‐PET images were acquired from 52 community‐dwelling Brazilians above 50 year old. Standardized uptake value ratio (SUVr) parametric maps were processed to a common 8 mm FWHM and generated using the pons as the reference region (Figure 1). We extracted the mean values of regions of interest using the ICBM152 atlas. [18F]FDG‐PET MBNs were constructed using a novel multiple sampling scheme, which assembles a stable group representative MBN based on bootstrap (n = 2000). Adaptive Synthetic Sampling Approach for Imbalance (ADASYN) was used to account for group imbalance and generated the ADA‐MBNs. Graph measures, including density, global efficiency, average degree, and assortativity coefficient were computed. Data were corrected for multiple comparisons using the False Discovery Rate (FDR) method (p<0.05).

**Result:**

41 individuals with long‐COVID and 11 healthy controls (HC) were included (Table 1). We observed that long‐COVID individuals present PET hyperconnectivity in both MBN and ADA‐MBN. (Figure 2a‐b). The long‐COVID group presented increased density, global efficiency and average degree whereas assortativity coefficient were reduced in both MBN and ADA‐MBN.

**Conclusion:**

Our findings showed that individuals with long‐COVID presented a brain metabolic hyperconnectivity, which is supported by increased density and average degree and may indicate a potential compensatory mechanism within the brain. In addition, the increase in global efficiency indicates that the brain of long‐COVID individuals exchanges metabolic information more efficiently, but the decreased assortativity coefficient suggests vertices with different properties connect to each other. Further longitudinal studies should follow these individuals for assessing microstructural and cognitive changes.